# Identification of Potential Genetic Biomarkers and Target Genes of Peri-Implantitis Using Bioinformatics Tools

**DOI:** 10.1155/2021/1759214

**Published:** 2021-12-11

**Authors:** Xiaogen Zhang, Zhifa Wang, Li Hu, Xiaoqing Shen, Chundong Liu

**Affiliations:** ^1^Department of Stomatology, Zhujiang Hospital, Southern Medical University, Guangzhou, Guangdong 510280, China; ^2^Department of Stomatology, General Hospital of Southern Theater of PLA, Guangzhou, Guangdong 510010, China

## Abstract

**Objectives:**

To investigate potential genetic biomarkers of peri-implantitis and target genes for the therapy of peri-implantitis by bioinformatics analysis of publicly available data.

**Methods:**

The GSE33774 microarray dataset was downloaded from the Gene Expression Omnibus (GEO). The differentially expressed genes (DEGs) between peri-implantitis and healthy gingival tissues were identified using the GEO2R tool. GO enrichment analysis and Kyoto Encyclopedia of Genes and Genomes (KEGG) pathway enrichment analysis were performed using the DAVID database and the Metascape tool, and the results were expressed as a bubble diagram. The protein-protein interaction network of DEGs was constructed using the Search Tool for the Retrieval of Interacting Genes (STRING) and visualized using Cytoscape. The hub genes were screened by the cytoHubba plugin of Cytoscape. The potential target genes associated with peri-implantitis were obtained from the DisGeNET database and the Open Targets Platform. The intersecting genes were identified using the Venn diagram web tool.

**Results:**

Between the peri-implantitis group and the healthy group, 205 DEGs were investigated including 140 upregulated genes and 65 downregulated genes. These DEGs were mainly enriched in functions such as the immune response, inflammatory response, cell adhesion, receptor activity, and protease binding. The results of KEGG pathway enrichment analysis revealed that DEGs were mainly involved in the cytokine-cytokine receptor interaction, pathways in cancer, and the *PI3K-Akt* signaling pathway. The intersecting genes, including *IL6*, *TLR4*, *FN1*, *IL1β*, *CXCL8*, *MMP9*, and *SPP1*, were revealed as potential genetic biomarkers and target genes of peri-implantitis.

**Conclusions:**

This study provides supportive evidence that *IL6*, *TLR4*, *FN1*, *IL1β*, *CXCL8*, *MMP9*, and *SPP1* might be used as potential target biomarkers for peri-implantitis which may provide further therapeutic potentials for peri-implantitis.

## 1. Introduction

In recent decades, dental implants have been widely used for the restoration of missing teeth with high success rates [1]. Peri-implantitis is a common complication of dental implants that can result in implant failure. Peri-implantitis can be defined as an inflammation of the peri-implant connective tissue and progressive loss of the supporting bone around the implants [2], and it is considered to be the leading cause of implant failure. According to previous literature, the approximate prevalence of peri-implantitis is 22% (range: 1%–47%) and that of peri-implant mucositis is 43% (range:19%–65%) [3, 4]. Studies have shown that bacterial infection could be the cause of the peri-implantitis and subsequent implant failure, and the various gene polymorphisms may be associated with the occurrence of peri-implantitis [2].

Although the underlying pathogenic mechanisms of peri-implantitis remain unclear, the excessive inflammatory response due to the microbial biofilms on implants and their toxins is believed to play an important role in the occurrence of peri-implantitis [5, 6]. Therefore, the immune-inflammatory response elicited by the bacterial biofilm may be responsible for the gingival recession and alveolar bone loss associated with peri-implantitis. Lipopolysaccharide can induce the cells of gingival and osseous tissues to overexpress proinflammatory cytokines including interleukin- (IL-) 1*β* and IL-6 [7]. Different methods have been used for treating peri-implantitis, such as mechanical debridement, implant surface modifications, adjunctive antibiotic therapy, and surgery. Gene therapy might be considered as a therapeutic option in regenerative medicine for peri-implant tissues [8].

In recent years, host modulation therapies are considered as a potential alternative for the treatment of peri-implantitis [9]. This treatment method based on the effects of inflammatory regulation not only promotes the efficacy of traditional management approaches for peri-implantitis but also reduces the risk of systemic disease and inflammation. Proinflammatory cytokines, such as IL-1*β*, IL-6, and TNF-*α*, have been used as biomarkers to identify periodontitis and peri-implantitis [10, 11]. There are also many other well-accepted biomarkers of tissue destruction and systemic inflammation, including matrix metalloproteinase- (MMP-) 8, MMP-9, high-sensitivity C-reactive protein, TNF-*α*, and IL-6, which are easily detected in both oral fluids and blood samples [12–15]. Certain cytokine inhibitors such as TNF-*α* antagonists and IL-1 receptor antagonists have exhibited anti-inflammatory effects in periodontal diseases and may be used for treating peri-implantitis [16, 17]. Further investigation of the underlying mechanism of peri-implantitis is needed to develop rational treatment strategies.

In recent years, bioinformatics methods have been widely used to analyze microarray data to identify differentially expressed genes (DEGs). Numerous bioinformatics tools and approaches have been developed, which could help us to better understand the underlying mechanisms [18–20]. Scientific literature on peri-implantitis has increased rapidly in recent years and particularly in the last 5 years [21]. However, few studies have focused on the application of bioinformatics analysis to gain insights on peri-implantitis. The study of Becker et al. showed that peri-implantitis and periodontitis show different mRNA signatures, although they share similar clinical characteristics [22]. In the present study, to better understand the potential molecular biomarkers and the potential therapeutic agents for peri-implantitis, we used the GSE33774 microarray dataset and bioinformatics tools. We downloaded mRNA expression profiles from Gene Expression Omnibus (GEO, http://www. http://ncbi.nlm.nih.gov/geo/). We identified the DEGs between samples of peri-implantitis and healthy samples. Gene Ontology (GO) is a major bioinformatics tool to annotate genes and analyze the biological process of these genes [23]. Kyoto Encyclopedia of Genes and Genomes (KEGG) is a widely used database that stores extensive data on genomes, biological pathways, diseases, chemical substances, and drugs. GO analysis is a common useful method for large-scale functional enrichment research, wherein gene functions can be classified into biological process (BP), molecular function (MF), and cellular component (CC). In the present study, KEGG pathway and GO functional enrichment analyses were performed. The DisGeNET platform and the Open Targets Platform (OTP) were used to further investigate the coexpressed genes associated with peri-implantitis. The findings of this study may help to predict the molecular mechanism and the potential therapeutic targets of peri-implantitis.

## 2. Materials and Methods

### 2.1. Data Source and Identification of DEGs

The gene expression dataset of GSE33774 analyzed in this study was obtained from the GEO database (http://www.ncbi.nlm.nih.gov/geo/) [24, 25]. GSE33774 was based on the Agilent GPL platform GPL6244 (Affymetrix Human Gene 1.0 ST Array (transcript (gene) version)). The GSE33774 dataset contains seven gingival tissue samples of peri-implantitis, seven gingival tissue samples of periodontitis, and eight healthy gingival samples [22]. All of the data are freely available online, and this study did not involve any experiments on humans or animals.

The DEGs between peri-implantitis samples and healthy samples were analyzed using the GEO2R online analysis tool (http://www.ncbi.nlm.nih.gov/geo/geo2r). The DEGs with the threshold criterion of ∣log(fold change) | ≥1.0 and *P* value < 0.05 were considered to be significantly differentially expressed.

### 2.2. GO and KEGG Pathway Enrichment Analyses of DEGs

To analyze the function of DEGs, biological analyses were performed using the online database DAVID [26, 27]. *P* < 0.05 was considered to be statistically significant. The Metascape tool (https://metascape.org) [28] was also used to perform functional and pathway enrichment analysis of DEGs, including BP, CC, MF, and KEGG pathway enrichment analysis. A *P* value of <0.05 was considered to be the cut-off criterion.

### 2.3. Protein-Protein Interaction (PPI) Network Construction and Hub Gene Identification

The PPI network of DEGs was constructed using the Search Tool for the Retrieval of Interacting Genes (STRING, http://string-db.org) (version 11.0) online database [29], and an interaction with a combined score of >0.40 (medium confidence interaction score) was considered to be statistically significant. Subsequently, the PPI network was visualized by Cytoscape software (version 3.8.0) [30]. Hub genes were identified and visualized using the CytoHubba plugin of Cytoscape [31]. The top ten genes with high degree of connectivity in the PPI network were identified as hub genes.

### 2.4. Prediction of the Gene-Disease Associations

To accurately predict the gene-disease associations of peri-implantitis, the DisGeNET platform (http://www.disgenet.org/) and the Open Targets Platform (OTP) (https://www.targetvalidation.org/) were used. DisGeNET is an online database that includes a collection of genes associated with human diseases based on expert-curated databases and scientific literature, and this database is publicly accessible [32]. The OTP provides evidence for human target-disease associations and tools that provide evidence-based systematic prioritization of targets for disease treatment [33]. The genes associated with peri-implantitis were exported from the DisGeNET database and the OTP. The following search terms were used in the DisGeNET database and the OTP (UMLS:C2936258) and peri-implantitis (EFO: 1001390), respectively. The intersecting genes of the top 10 hub genes and the disease-associated genes obtained from the DisGeNET database and the OTP were identified using the Venn diagram web tool (bioinformatics.psb.ugent.be/webtools/Venn/). A gene-disease network around peri-implantitis was generated by the DisGeNET Cytoscape plugin.

## 3. Results

### 3.1. Identification of DEGs

**Figure 1:**
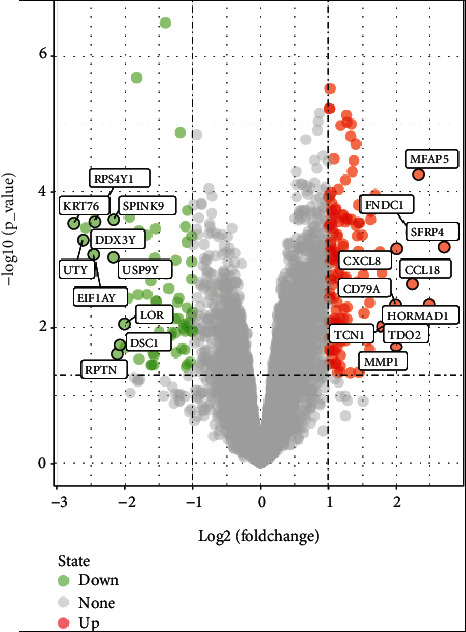
Volcano maps of differentially expressed genes. Red and green spots represent differentially expressed genes: red spots represent upregulated genes and green spots represent downregulated genes.

The microarray expression profile of GSE33774 was selected in this study. We identified 205 DEGs including 140 upregulated genes and 65 downregulated genes based on the criteria of *P* < 0.05 and ∣logFC  | ≥1.0. All DEGs were identified by comparing samples of peri-implantitis with healthy gingival samples. Subsequently, the volcano plots were generated for the identified DEGs (Figure 1).

### 3.2. Functional Enrichment Analysis of DEGs

**Figure 2:**
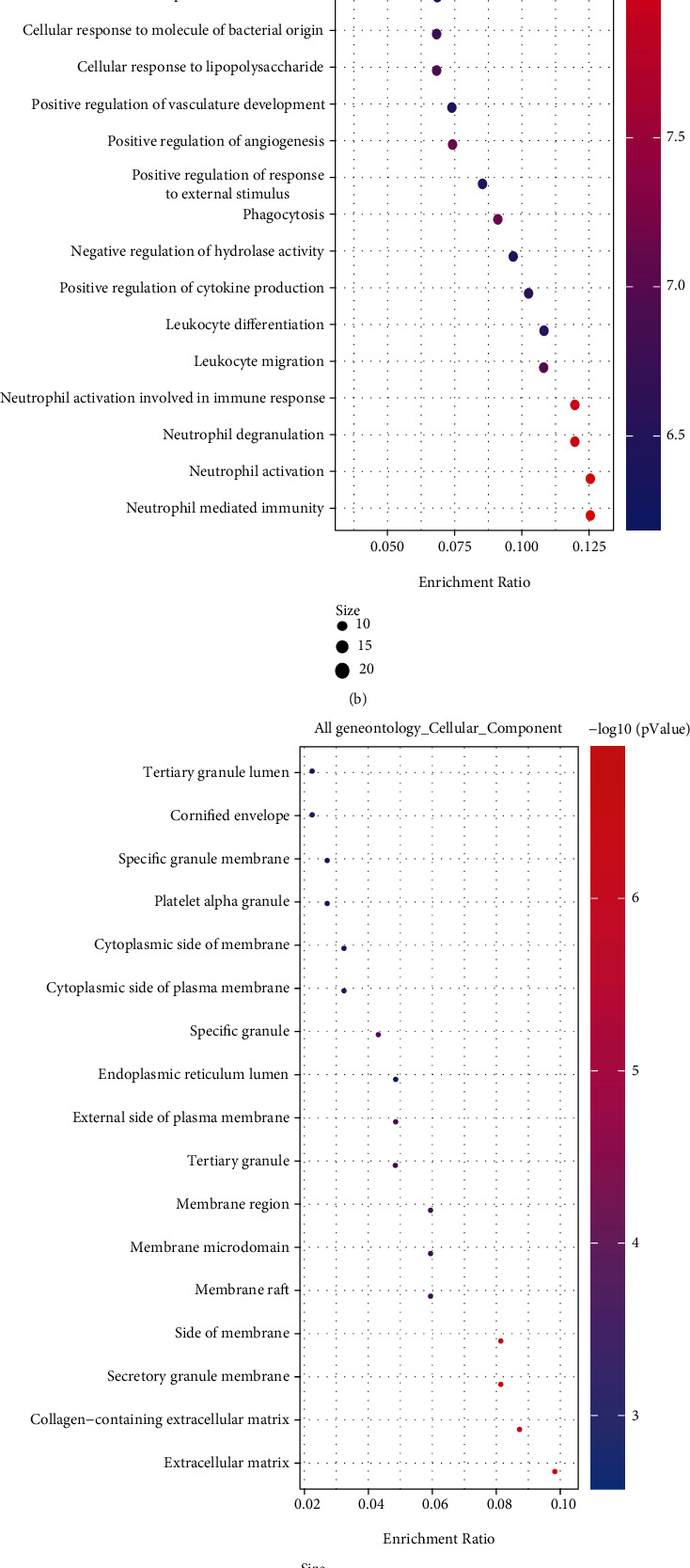
Results of GO and KEGG analysis results of differentially expressed genes: (a) KEGG pathway enrichment results; (b) GO biological process enrichment results; (c) GO cell component enrichment results; (d) GO molecular function enrichment results. The *x*-axis represents gene ratio, and the *y*-axis represents GO terms. The size of each circle indicates gene count. The color of circles represents different -log10(*P*.values).

**Figure 3:**
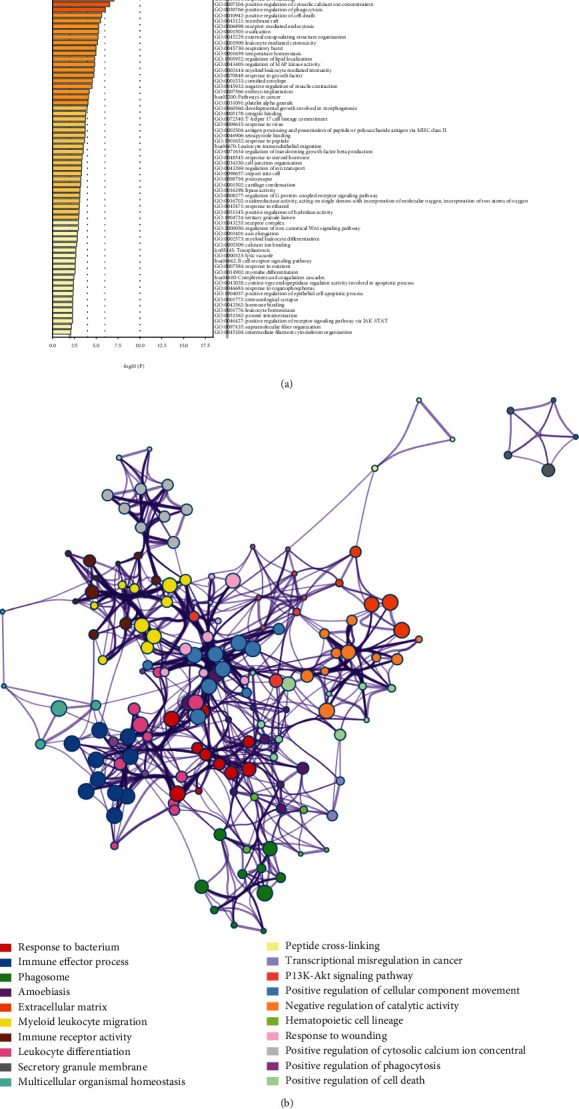
DEG enrichment results obtained using Metascape: (a) significantly enriched GO terms and KEGG pathways of DEGs; (b) network contact of GO terms and KEGG pathways.

GO enrichment analysis and the KEGG pathway enrichment analysis of DEGs were performed using the online database DAVID (Table 1). The enriched GO terms were divided into BP, CC, and MF ontologies. The results indicated that for BP analysis, the DEGs were mainly enriched in immune response, inflammatory response, signal transduction, and cell adhesion. For the CC terms, the DEGs were enriched in plasma membrane, integral component of membrane, extracellular exosome, extracellular space, and extracellular region. The MF analysis showed that the DEGs were enriched in receptor activity, protease binding, RNA polymerase II regulatory region sequence-specific DNA binding, and transmembrane signaling receptor activity. The results of the KEGG pathway enrichment analysis showed that DEGs were mainly enriched in cytokine-cytokine receptor interaction, pathways in cancer, amoebiasis, phagosome, and the PI3K-Akt signaling pathway. The results obtained by enrichment analysis were illustrated by a bubble diagram (Figure 2). The results of the enrichment analysis of DEGs performed by Metascape are shown in Figure 3. DEGs are mainly concentrated in response to the bacterium and immune effector process. DEGs associated with innate immune responses and defense responses may play an important role in inflammation associated with peri-implantitis.

### 3.3. Analysis of the PPI Network and Identification of Hub Genes

**Figure 4:**
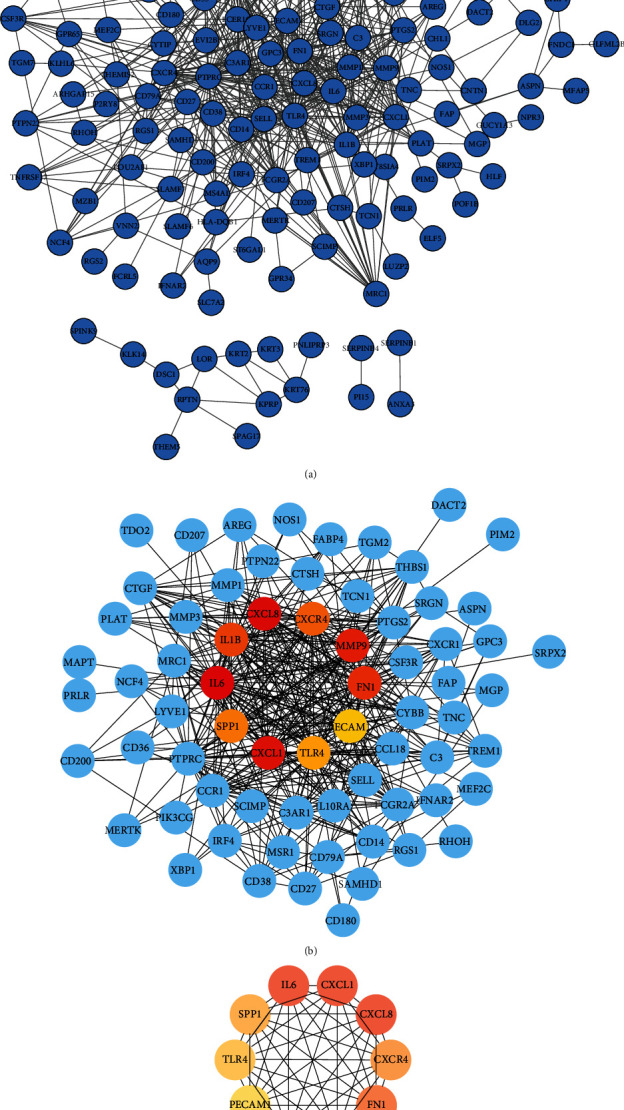
(a) Protein-protein interaction network of DEGs. (b) The top 10 hub genes in the PPI network with neighbors and expanded network. (c) Subnetwork of the top 10 hub genes from the PPI network.

Protein interactions among the DEGs were predicted with STRING tools. A total of 143 nodes and 601 edges were involved in the PPI network, as shown in Figure 4(a). The top 10 genes according to their degree of connectivity in the PPI network were identified as hub genes (Figures 4(b) and 4(c)). The results showed that *IL6*, *TLR4*, *FN1*, *IL1β*, *MMP9*, *CXCL8*, *CXCR4*, *CXCL1*, *PECAM1*, and *SPP1* were identified as hub genes (Table 2). Among these genes, IL-6 and TLR4 showed the highest node degrees, suggesting that they may play important roles in peri-implantitis. All the 10 hub genes were upregulated in peri-implantitis. As shown in Figure 4(c), all hub genes interact with each other directly. The hub genes were closely related to the results of GO and KEGG pathway enrichment analyses (Table 3). As shown in Table 3, CXCL8, CXCL1, IL-6, IL-1*β*, and TLR4 are involved in the immune response (GO:0006955), while CXCL8, CXCR4, CXCL1, IL-6, IL-1*β*, SPP1, and TLR4 are involved in the inflammatory response (GO:0006954). CXCL8, CXCL1, IL-6, and IL-1*β* are directly involved in the cytokine-cytokine receptor interaction pathway (hsa04060).

### 3.4. The Potential Target Genes Associated with Peri-Implantitis

**Figure 5:**
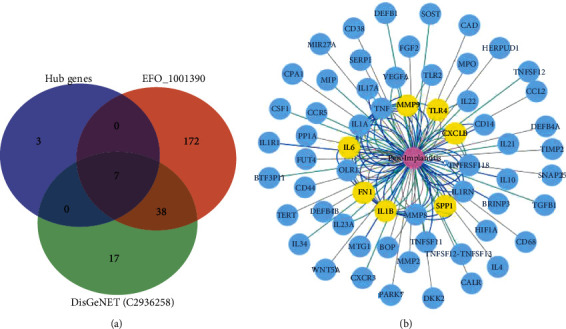
(a) Venn diagram of the intersecting genes of the top 10 hub genes and the associated genes obtained from the DisGeNET database (UMLS: C2936258) and the OTP (EFO: 1001390). (b) The gene-disease networks generated using the DisGeNET Cytoscape plugin, yellow nodes represent the intersecting genes.

Sixty-two target genes associated with peri-implantitis were downloaded from the DisGeNET database, and 217 potential target genes were obtained from the OTP. The intersecting genes, including IL-6, TLR4, FN1, IL-1*β*, CXCL8, MMP-9, and SPP1, were identified using the Venn diagram web tool (Figure 5(a)). The gene-disease network around the peri-implantitis was generated by using the DisGeNET Cytoscape plugin (Figure 5(b)).

## 4. Discussion

In the present study, bioinformatics methods were used to analyze the critical genes and pathways that were associated with peri-implantitis using the GSE33774 microarray and bioinformatics tools. By using the GSE33774 microarray dataset, Becker et al. found that peri-implantitis and periodontitis show different mRNA signatures [22]. In the present study, we investigated the DEGs between seven gingival tissue samples of peri-implantitis and eight healthy gingival samples by using the GSE33774 dataset. We examined a total of 13,057 DEGs, of which 205 DEGs were considered for further studies and 10 hub genes were identified. Potential disease-related genes were collected from the DisGeNET database and the OTP. The DisGeNET database has been used to study a variety of biomedical issues, and it contains one of the largest publicly available collections of genes and variants related to human diseases for investigating the molecular basis of specific diseases [32]. The OTP provides disease-centric or target-centric workflows that facilitate target selection and validation [33]. The validity was verified by intersecting the hub genes with the potential target genes obtained from the DisGeNET and OTP. Finally, the potential target genes, namely, IL-6, TLR4, FN1, IL-1*β*, CXCL8, MMP-9, and SPP1, were found to be associated with peri-implantitis, and our results were consistent with those of previous studies [16, 34].

A previous study showed that osteoclastogenesis-related cytokines may be associated with the occurrence and the severity of peri-implantitis [35]. Moreover, the proinflammatory cytokines, such as IL-1*β*, IL-6, and TNF-*α*, have been used as biomarkers to diagnose periodontitis and peri-implantitis [11]. Analysis of cytokine levels may help to confirm the early diagnosis of peri-implantitis in high-risk patients. In vitro experiments have shown that the levels of proinflammatory cytokines increase in peri-implantitis, and these levels significantly decrease after clinical treatment [34]. In the present study, our results showed that the proinflammatory cytokines IL-6, IL-1*β*, and CXCL8 were upregulated in peri-implantitis. The monitoring of TNF-*α*, CXCL8, and IL-1*β* levels could be considered as one of the diagnostic elements [36].

For the BP terms, the DEGs were enriched in the immune response and inflammatory response, and this result was consistent with that of previous studies [37]. For the CC terms of GO, the DEGs were enriched in the integral component of the membrane, which included 68 DEGs. In the MF analysis, the DEGs were the most significantly enriched in the immune response, inflammatory response, signal transduction, and cytokine-cytokine receptor interaction pathway. The hub genes play an important role in understanding the biological mechanism of peri-implantitis. The hub genes were closely related to the results of GO and KEGG pathway enrichment analyses as shown in Table 3. Our findings showed that the CXCL8, IL-6, IL-1*β*, and TLR4 genes are related to immune response (GO:0006955) and inflammatory response (GO:0006954). The CXCL8, IL-6, and IL-1*β* genes are directly involved in the cytokine-cytokine receptor interaction pathway (hsa04060). The hub gene IL-6 is a cytokine that stimulates immune response and is upregulated in peri-implantitis [37]. SPP1 is also known as OPN, and it is a type of osteoimmunoinflammatory marker related to the inflammation and regulation of cytokine production [17].

Deng et al. [38] found that TLR4 signaling may mediate inflammation and bone resorption in peri-implantitis through the regulation of B cell infiltration, the RANKL/OPG ratio, and differential inflammatory cytokine production. Previous studies have shown that the anti-inflammatory microRNA miR-146a enhances the inhibition of peri-implant bone resorption through the regulation of TLR2/4 signaling [39] and Wnt5a involved in TLR4 signaling induces the production of inflammatory cytokines and causes breakdown of extracellular matrix in peri-implantitis [40]. The microRNAs miR-146a and miR-146b are the most common members of the miR-146 family in periodontitis lesions, and miR-146a may protect gingival tissue from immune-mediated periodontal inflammation [41]. Correspondingly, the KEGG pathway analysis showed that these DEGs were mapped to cytokine-cytokine receptor interaction, pathways in cancer, amoebiasis, phagosome, and the PI3K-Akt signaling pathway, all of which were consistent with the results of previous studies.

Regarding the other target genes, the expression level of FN1 can reflect the progress of periodontitis or peri-implantitis. The mRNA expression of FN1 in the peri-implantitis group was significantly higher than that in the control group [42]. Cellular fibronectin occurs abundantly in the periodontium and may be associated with the state of implants [40]. MMP-9 is involved in the progression of peri-implantitis and is correlated with LOX-1 and the ERK1/2-mediated signaling pathway [43]. However, the regulatory mechanisms of MMP-9 in peri-implantitis need to be well elucidated. The LOX-1/MMP-9 signaling pathway and OPN may be potential drug targets to decrease the levels of proinflammatory cytokines and increase apoptosis in peri-implantitis [37, 43].

Host modulation therapy with anti-inflammatory drugs has been used as a potential method for treating periodontitis [44]. Based on the present literature, many immunoinflammatory molecules can be considered as potential biomarkers for diagnosis of peri-implantitis [45].

## 5. Conclusion

Our bioinformatics analysis identified 205 DEGs between gingival tissues of peri-implantitis and healthy tissues based on the gene expression datasets obtained from the GEO database, and the potential therapeutic target genes were validated by the analysis of the DisGeNET database and the OTP. We found that IL-6, TLR4, FN1, IL-1*β*, CXCL8, MMP-9, and SPP1 might be used as potential biomarkers for the diagnosis of peri-implantitis. Further studies are needed to reveal the potential association of these genes with peri-implantitis and to determine potential therapeutic drug targets for peri-implantitis.

## Figures and Tables

**Table 1:** The results of GO function analysis and KEGG pathway enrichment analysis (top 5 terms are listed).

Category	Term	Count	*P* value
BP term	GO:0006955~immune response	23	4.62*E* − 10
BP term	GO:0006954~inflammatory response	20	1.51*E* − 08
BP term	GO:0007165~signal transduction	20	0.032080
BP term	GO:0007155~cell adhesion	19	1.38*E* − 06
BP term	GO:0007186~G-protein-coupled receptor signaling pathway	17	0.024583
CC term	GO:0016021~integral component of membrane	68	0.008933
CC term	GO:0070062~extracellular exosome	43	0.004559
CC term	GO:0005615~extracellular space	40	1.38*E* − 09
CC term	GO:0005576~extracellular region	38	1.42*E* − 06
MF term	GO:0004872~receptor activity	11	7.84*E* − 05
MF term	GO:0004252~serine-type endopeptidase activity	10	0.001235
MF term	GO:0002020~protease binding	7	5.84*E* − 04
MF term	GO:0000977~RNA polymerase II regulatory region sequence-specific DNA binding	7	0.019798
MF term	GO:0004888~transmembrane signaling receptor activity	7	0.022448
KEGG pathway	hsa04060:cytokine-cytokine receptor interaction	14	8.47*E* − 06
KEGG pathway	hsa05200:pathways in cancer	12	0.009391
KEGG pathway	hsa05146:amoebiasis	11	6.57*E* − 07
KEGG pathway	hsa04145:phagosome	11	1.54*E* − 05
KEGG pathway	hsa04151:PI3K-Akt signaling pathway	11	0.010471

**Table 2:** Top 10 hub genes with higher degree of connectivity.

Gene symbol	Gene description	Degree
IL-6	Interleukin-6	46
TLR4	Toll-like receptor 4	43
FN1	Fibronectin 1	33
IL-1*β*	Interleukin-1 beta	32
CXCL8	C-X-C motif chemokine ligand 8	32
MMP-9	Matrix metallopeptidase-9	31
CXCR4	C-X-C motif chemokine receptor 4	30
CXCL1	C-X-C motif chemokine ligand 1	27
PECAM1	Platelet and endothelial cell adhesion molecule 1	26
SPP1	Secreted phosphoprotein 1	22

**Table 3:** Top 10 hub genes that were closely associated with the results of GO and KEGG analyses.

Term	Genes
GO:0006955~immune response	CCR1, XBP1, **CXCL8**, GPR65, AQP9, NCF4, **CXCL1**, SERPINB9, SAMHD1, THBS1, LAX1, C3, **IL-6**, IGKC, RGS1, **IL-1*β***, PXDN, ENPP2, CD27, CD36, CCL18, **TLR4**, HLA-DQB1
GO:0006954~inflammatory response	CCR1, **CXCL8**, CD180, CYBB, **CXCR4**, **CXCL1**, PTGS2, THBS1, PIK3CG, C3, **IL-6**, THEMIS2, CXCR1, **IL-1*β***, **SPP1**, C3AR1, CD27, CD14, CCL18, **TLR4**
GO:0007165~signal transduction	CD53, GABRP, SH3GL3, CSF3R, **CXCL8**, **CXCL1**, ABCC9, HTR3A, ARHGAP15, LYVE1, C3, EVI2A, CHL1, RGS1, **IL-1*β***, MRC1, **PECAM1**, TNFRSF17, CD38, CCL18
GO:0007155~cell adhesion	NLGN4Y, CCR1, CSF3R, LAMB4, TNC, **FN1**, LYVE1, THBS1, CTGF, THEMIS2, SELL, FAP, CHL1, BOC, **SPP1**, **PECAM1**, CNTN1, SLAMF7, CD36
GO:0007186~G-protein-coupled receptor signaling pathway	**CXCL8**, GPR34, GPR65, **CXCR4**, **CXCL1**, AREG, PIK3CG, C3, SFRP4, SFRP2, CXCR1, RGS1, C3AR1, ENPP2, CCL18, ADGRL4, TGM2
GO:0005886~plasma membrane	CSF3R, GPR65, AQP9, SLC2A3, TREM1, PIK3CG, CTGF, RGS5, RGS2, CHL1, RGS1, BOC, MRC1, ENPP2, C3AR1, CD38, CD36, LRRFIP1, TGM2, GUCY1A3, CD53, IFNAR2, FCER1G, FCRL5, ANXA3, DCC, CYBB, RHOH, HTR3A, ABCC9, PRLR, LAX1, CDHR1, **PECAM1**, SLC27A6, PLIN2, MAPT, DSC1, **TLR4**, HLA-DQB1, SLC47A2, NPR3, KCNA3, **CXCR4**, SAMHD1, SLC7A2, RASGRP3, P2RY8, C3, CD79A, IGKC, CXCR1, GPC3, SLAMF7, TNFRSF17, SLAMF6, CD14, CCR1, MSR1, GABRP, IL-10RA, MERTK, LYVE1, PTPRC, FCGR2A, DLG2, SELL, FAP, VNN2, CD207, CNTN1, CD27, ADGRL4, CDK14, F2RL2, CD200
GO:0016021~integral component of membrane	CSF3R, DENND5B, GPR65, AQP9, LRMP, SLC2A3, TREM1, AREG, MS4A4A, AADACL2, CHL1, ENPP2, C3AR1, CD38, CD36, UTY, GUCY1A3, CD53, IFNAR2, ST6GAL1, FCER1G, GPR34, FCRL5, DCC, CYBB, HTR3A, ABCC9, PRLR, LAX1, SFRP4, SFRP2, CDHR1, **PECAM1**, SLC27A6, CFAP54, DSC1, HLA-DQB1, SLC47A2, NPR3, **CXCR4**, SEL1L3, P2RY8, CD79A, CXCR1, SLITRK3, SLAMF7, TNFRSF17, SLAMF6, CCR1, MSR1, GABRP, XBP1, IL-10RA, MERTK, LYVE1, SCIMP, TMEM156, PTPRC, FCGR2A, EVI2A, SELL, FAP, CD207, ESYT3, EVI2B, ADGRL4, F2RL2, CD200
GO:0070062~extracellular exosome	KPRP, SH3GL3, NPR3, **CXCR4**, PLAT, SLC2A3, THBS1, C3, IGKC, CHL1, GPC3, **SPP1**, CTSH, CD38, SLAMF6, CD14, TGM2, CD53, SERPINB4, SERPINB1, ST6GAL1, KRT3, ANXA3, KRT2, **FN1**, KLK14, SERPINB9, KRT76, LYVE1, **MMP-9**, ALDH1A3, PTPRC, FCGR2A, FABP4, **IL-1*β***, PXDN, MGP, PI15, **PECAM1**, CNTN1, CD27, DSC1, MS4A1
GO:0005615~extracellular space	**CXCL8**, TNC, PLAT, **CXCL1**, THBS1, AREG, CTGF, C3, SRPX2, IGKC, GPC3, **SPP1**, ENPP2, CTSH, CD14, CD36, CCL18, NLGN4Y, SRGN, IFNAR2, SERPINB4, SERPINB1, KRT2, MMP-3, **FN1**, KLK14, SERPINB9, IGFL1, MERTK, **MMP-9**, SFRP4, **IL-6**, SFRP2, FAP, TCN1, **IL1-*β***, PXDN, **PECAM1**, MS4A1, BPIFC
GO:0005576~extracellular region	CSF3R, OLFML2B, **CXCL8**, LUZP2, TNC, PLAT, **CXCL1**, TREM1, THBS1, CTGF, C3, AADACL2, IGKC, **SPP1**, LIPM, LIPK, CD14, SRGN, IFNAR2, MMP-1, MMP-3, **FN1**, **MMP-9**, PRLR, MFAP5, GZMK, SFRP4, **IL-6**, SFRP2, FNDC1, TCN1, **IL-1*β***, MZB1, CD27, PLIN2, SPINK9, F2RL2, PNLIPRP3
GO:0004872~receptor activity	GUCY1A3, NLGN4Y, CSF3R, IL-10RA, CD180, MRC1, TNFRSF17, TREM1, **TLR4**, LYVE1, CD200
GO:0004252~serine-type endopeptidase activity	GZMK, C3, IGKC, FAP, MMP-1, MMP-3, KLK14, CTSH, PLAT, **MMP-9**
GO:0002020~protease binding	SERPINB4, XBP1, SELL, FAP, CHL1, **FN1**, SERPINB9
GO:0000977~RNA polymerase II regulatory region sequence-specific DNA binding	MEF2C, XBP1, HLF, ELF5, EAF2, ZFY, BARX2
GO:0004888~transmembrane signaling receptor activity	CD79A, EVI2A, DCC, MRC1, CD27, **TLR4**, LYVE1
hsa04060:cytokine-cytokine receptor interaction	CCR1, IFNAR2, CSF3R, **CXCL8**, IL-10RA, **CXCR4**, **CXCL1**, PRLR, **IL-6**, CXCR1, **IL-1*β***, TNFRSF17, CD27, CCL18
hsa05200:pathways in cancer	**IL-6**, CSF3R, **CXCL8**, MMP-1, DCC, LAMB4, **FN1**, **CXCR4**, PTGS2, **MMP-9**, PIK3CG, RASGRP3
hsa05146:amoebiasis	SERPINB4, **IL-6**, SERPINB1, **CXCL8**, **IL-1*β***, LAMB4, **FN1**, SERPINB9, CD14, **TLR4**, PIK3CG
hsa04145:phagosome	C3, MSR1, FCGR2A, NCF4, MRC1, CD14, CD36, NOS1, THBS1, **TLR4**, HLA-DQB1
hsa04151:PI3K-Akt signaling pathway	IFNAR2, **IL-6**, CSF3R, LAMB4, **SPP1**, TNC, **FN1**, THBS1, PRLR, **TLR4**, PIK3CG

## Data Availability

The datasets analyzed during the current study are available in the Gene Expression Omnibus with the accession GSE33774.

## Abbreviations

GEO: Gene Expression Omnibus
DEGs: Differentially expressed genes
GO: Gene Ontology
KEGG: Kyoto Encyclopedia of Genes and Genomes
DAVID: The Database for Annotation, Visualization, and Integrated Discovery
BP: Biological process
MF: Molecular function
CC: Cellular component
PPI: Protein-protein interaction
STRING: Search Tool for the Retrieval of Interacting Genes Database
OTP: The Open Targets Platform
IL-6: Interleukin-6
TNF-*α*: Tumor necrosis factor *α*
TLR4: Toll-like receptor 4
FN1: Fibronectin 1
IL-1*β*: Interleukin 1 beta
CXCL8: C-X-C motif chemokine ligand 8
MMP-9: Matrix metallopeptidase-9
SPP1: Secreted phosphoprotein 1
CXCL1: C-X-C motif chemokine ligand 1
PECAM1: Platelet and endothelial cell adhesion molecule 1
MMP-8: Matrix metalloproteinase-8.
